# Myopia management functional lenses (MMFL): a bibliometric analysis of multidisciplinary perspective and trend insights in the context of vision health

**DOI:** 10.1007/s10792-026-04126-3

**Published:** 2026-06-29

**Authors:** Ling Wang, Qing Zhang, Zhi-Chao Jia, Lei Ji, Jun Huang

**Affiliations:** 1 Department of Optometry Engineering, Jinling Institute of Technology, Nanjing, 211169 China; 2 Ophthalmology Department, Nanjing Vision Eye Hospital, Nanjing, 210000 China

**Keywords:** Myopia management, Spectacle lens, Contact lens, Visual analysis, Bibliometrics

## Abstract

**Purpose:**

To analyze the research dynamics of myopia management functional lenses (MMFL) in the field of vision health over the past decade, revealing cutting-edge hotspots and development trends.

**Methods:**

Relevant literature on MMFL from 2016 to 2025 in the Web of Science (WOS) core database was searched by computer. The bibliometrics software Bibliometrix, VOSviewer 1.6.20 and CiteSpace.V.6.3.R1 were used for bibliometric and knowledge graph visualisation analyses.

**Results:**

A total of 1143 WOS core database documents were included, and the number of publications has gradually increased over time. China and the United States rank the top two in terms of the number of publications and citations. The leading authors in this field are Cho P, Sankaridurg P, and Chen H, who have made significant contributions to research in two distinct subfields: contact lenses and spectacle lenses. Most relevant sources are OPHTHALMIC AND PHYSIOLOGICAL OPTICS, CONTACT LENS & ANTERIOR EYE, and OPTOMETRY AND VISION SCIENCE, all of which are considered to be highly authoritative publications in this field. These journals have published a considerable number of articles on a wide range of topics, including the mechanism of spectacle lenses, physiological changes associated with contact lenses, variations in fitting parameters, and the effects of myopia control. The keywords co-occurrence, clustering, thematic map, timeline view and emergent analyses reveal that recent research has been focused on the following areas: axial length, defocusing mechanism, choroidal thickness, and orthokeratology.

**Conclusion:**

The knowledge map of research on MMFL is constructed through bibliometric analysis, systematically summarizing the current status and hotspots of research. The integration of material, optics and intelligence is a trend that is set to be reflected in future functional lenses for myopia management.

## Introduction

The increasing prevalence of myopia is a growing global public health problem more than 90% of people with vision impairment have a preventable or treatable cause with existing highly cost-effective interventions [1]. Functional lens intervention is a key component in vision health. In particular, it is an essential tool for optometrists in managing myopia. China is currently experiencing a significant and potentially worsening epidemic of childhood myopia [2]. In terms of correcting myopia and ameliorating its occurrence, significantly increases the risk of visual impairment due to myopia-related ocular morbidity. Comprehensive interventions and management are needed to slow its progression during childhood and adolescence myopia management functional lenses (MMFL) as an optical drug, which contact lenses with their cosmetic advantages, and spectacle lenses with their ease of fitting and fewer complications, have gained popularity in recent years. However, there is still a lack of bibliometric analysis of the current status and hot trends of research, this is a first bibliometric literature exploration and analysis based on this domain. Moreover, the study will explore the future trend of MMFL according to the needs of vision health, and explore the direction of improvement and specific measures under the intersection of multiple disciplines.

## Data and methods

### Search strategy

The study data were obtained from the WOS core database using the advanced search function in WOS with the search strategy formula:

((TS = ("myopia control" OR "myopia management" OR "myopia progression" OR "myopia inhibit*" OR "slow* myopia" OR "delay* myopia")) AND TS = ((lens* OR spectacle* OR glass* OR "optical device*") OR ("contact lens*" OR orthokeratology OR ortho-k OR OKR))) OR TS = ((("defocus incorporat*" OR "DIMS" OR "HAL" OR "defocus" OR "multifocal" OR "extended depth of focus" OR EDOF OR "extended focus" OR "peripheral defocus") AND (lens* OR spectacle* OR "contact lens*")) AND (myopia OR nearsight*))

OKR = Optokinetic response, HAL = Highly aspherical lenslets, DIMS = Defocus Incorporated Multiple Segments, EDOF = Extended Depth of Focus

**Fig. 1:**
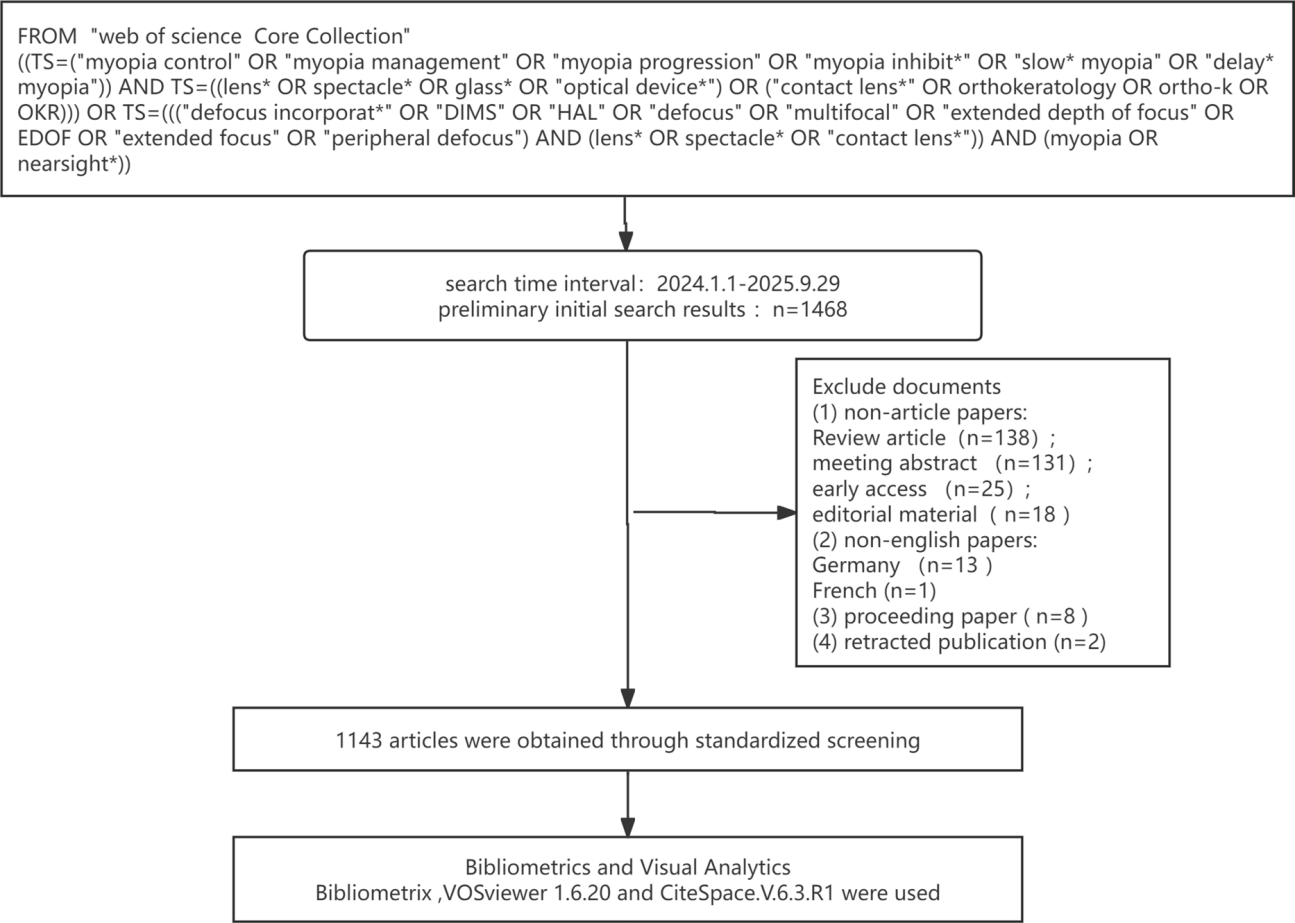
Flowchart of literature search

Retrieved on 2025.9.29. The time span was 2016–2025, the search language was English, and the type of literature was research papers, resulting in the inclusion of 1143 documents. The literature search process is shown in Fig. 1.

### Literature analysis

The search results were downloaded in plain text format with complete records and citations in the WOS core database. After data screening, we constructed country/region, institution and author collaboration network time-series overlay visualization maps through VOSviewer 1.6.20 [3], and simultaneously generated author citation analysis and publication source analysis and keyword co-occurrence network, to completely show the trend of various types of collaborations over time; used CiteSpace 6.3.R1 to perform clustering analysis and emergence intensity detection of keywords [4]. We used Bibliometrix [5] to generate country -author- journal three field plot to quantify the knowledge dissemination paths of core journals and cross-regional cooperation associations. Cluster analysis enables the categorisation of references and keywords, as well as the discovery of essential study topics. Bursts of keywords and references are frequently utilised to discover new research trends.

## Results

### Analysis of annual publications

**Fig. 2:**
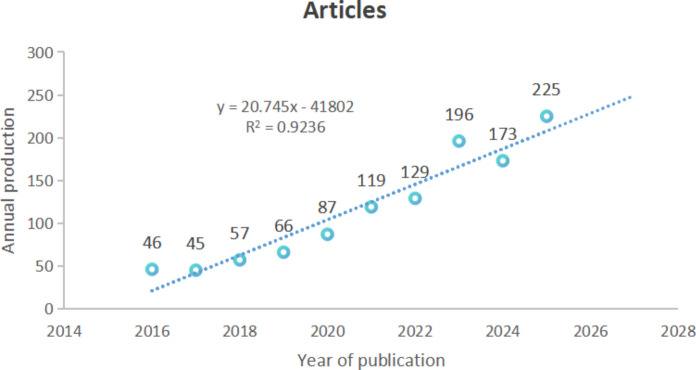
Annual publications of MMFL studies

A total of 1143 research papers on MMFL field were included, with a total of 20,242 citations, averaging 17.71 citations per paper. As can be seen in Fig. 2, the annual number of publications and the annual cumulative number of publications in this field show a steady upward trend, with an annual growth rate 19.29%. The number of publications is from 46 in 2016 to 225 in 2025. This is an increase of about 5 times. As shown in Fig. 2, there is a linear trend in the growth of annual production (R^2^ = 0.9236).

### Analysis of national/regional collaborative networks

**Fig. 3:**
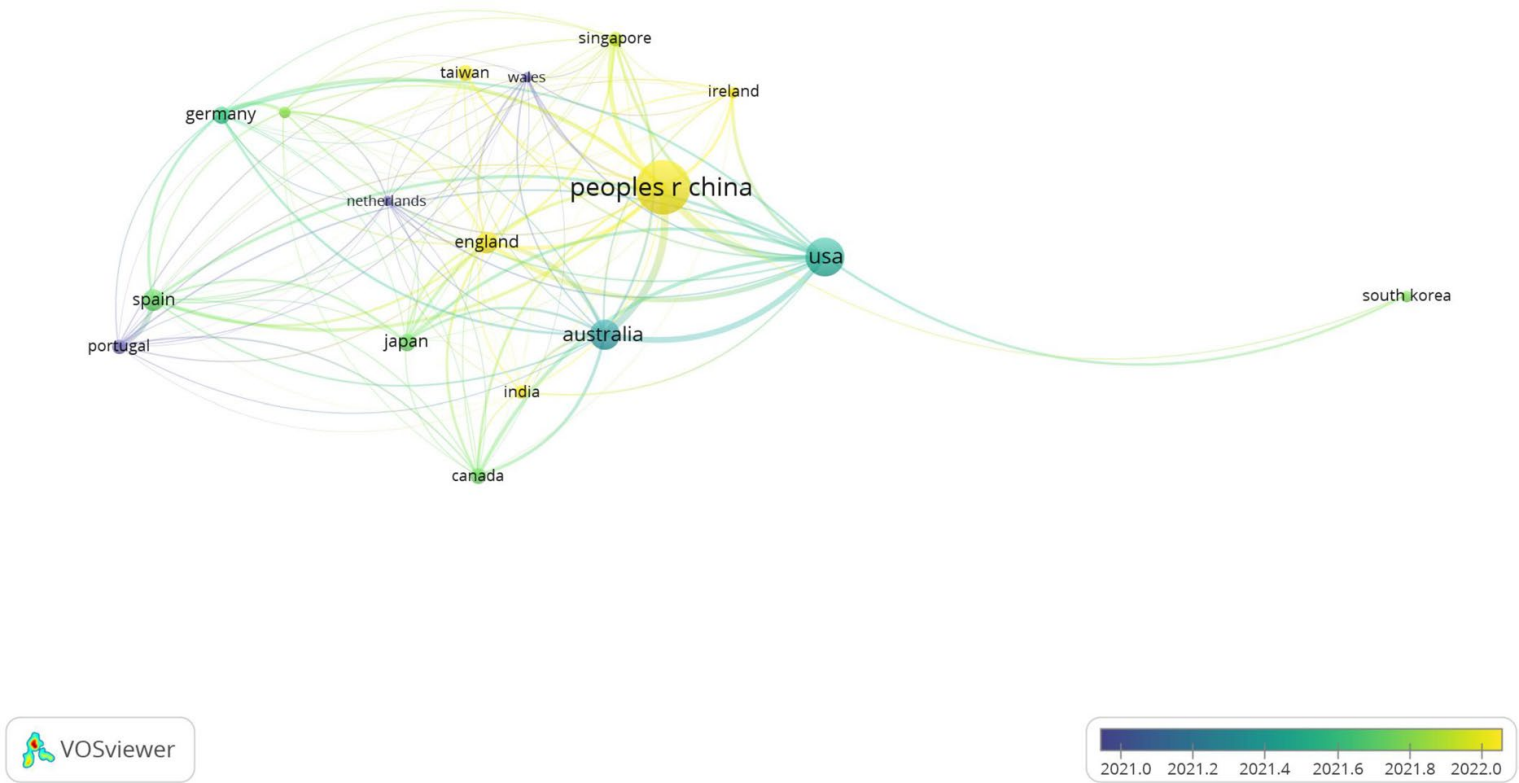
Time overlay visualization of national/regional collaborative networks node sizes representing the number of publications, the thickness of the lines indicating the collaborative relationships and their strengths, and the warm color of the nodes indicating a later average publication time

Figure 3 presents a overlay visualization of national/regional collaborative networks in the field of MMFL, presenting the collaborative relationships between countries/regions and their average publication times. The minimum publication threshold of VOSviewer sending countries was set to 15, and a time overlay graph of the collaboration network containing the top 17 research countries was included with The U.S. has made outstanding contributions to early research in this field with an early average publication time, while China has recently published a large number of academic results. As shown in the Fig. 3, China has the highest number of publications and more recent releases in the field, while and maintains close cooperation with countries such as the United States, the United Kingdom, and Australia. In addition, the top 10 countries in terms of publication volume and their related information, in which China (549 articles) accounts for the first place in the total publication volume. In terms of total citations, the United States is ranked second; also, the United States is ranked second in terms of total link strength total link strength refers to the number of times a country appears alongside other partner countries.

In terms of the number of publications and cooperative network relationship between countries, China is the country with the largest number of publications in the past 10 years, compared with the United States, the United Kingdom, and Australia, which are not as large as China in terms of the number of publications; however, these countries participated in the research at an earlier stage, and the citation counts of their classic literature and innovative research are higher, thus having a higher average number of citations per article. Therefore, China needs to further improve the quality and originality of its research and strengthen international cooperation.

### Analysis of affiliations

**Fig. 4:**
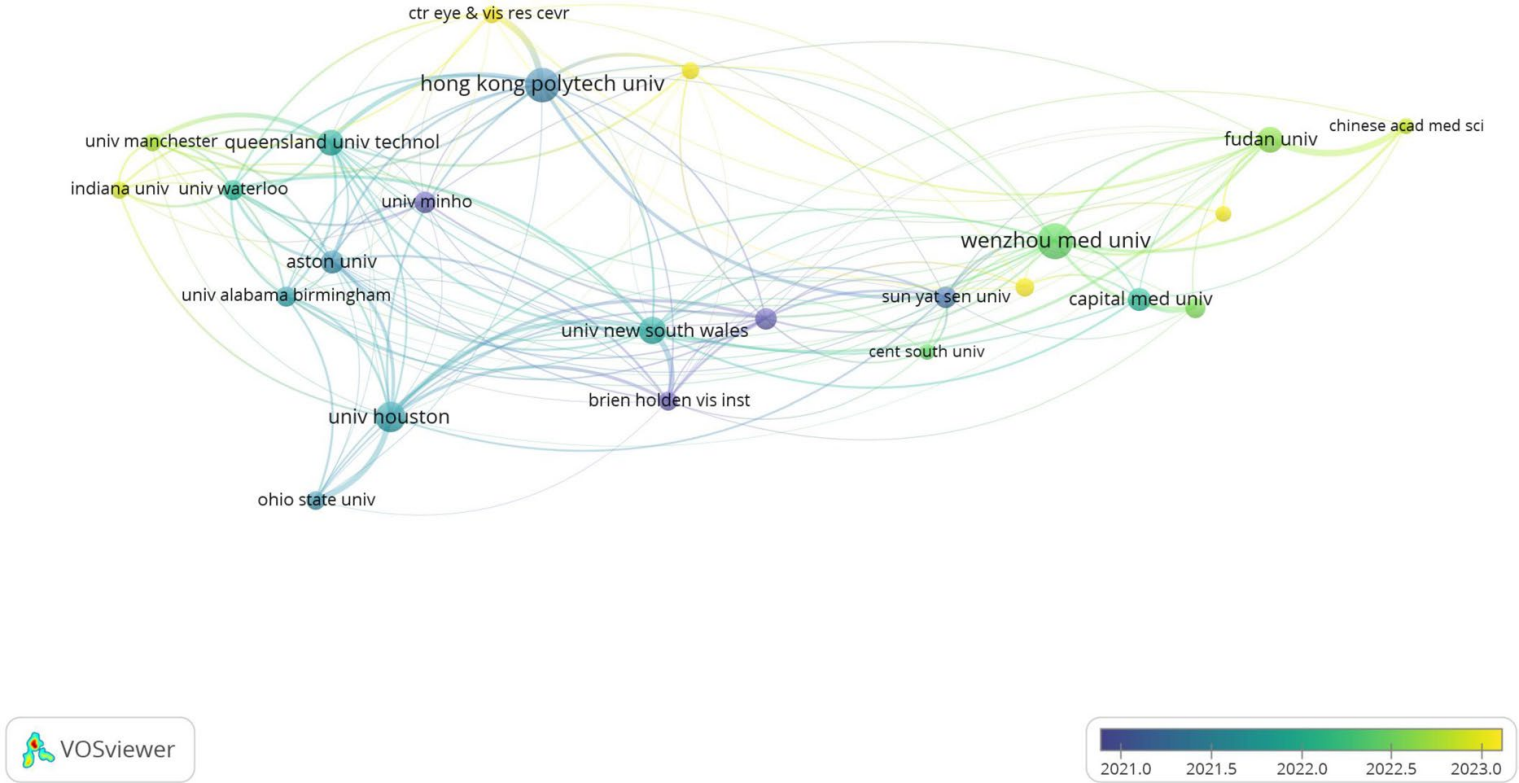
Time overlay visualization of optometric affiliations collaborative networks. The size of the spheres in the graph is proportional to the number of publications, the thickness of the connecting lines indicates the intensity of cooperation between institutions, and the color shade of the spheres reflects the average publication time of the institutions

The data show that a total of 1173 affiliations are involved in research related to this field. The minimum publication threshold of VOSviewer sending institutions was set to 20, and a time overlay graph of the collaboration network containing the top 24 research institutions was included (Fig. 4).

Table 1 lists the top 10 affiliations in terms of the number of publications. The Fig. 4 and the Table 1 results of the analysis showed that universities such as University California, Berkeley had an earlier volume of publications, while the volume of publications from various optometric affiliations in China has increased significantly in recent years.

**Table 1:** The top 10 productive affiliations

Rank	Institutions	Country	Documents	Citations	Total link strength
1	Wenzhou Medical University	China	95	2683	53
2	The Hong Kong Polytechnic University	China	91	3314	67
3	University of Houston	USA	67	3531	54
4	The University of New South Wales	China	58	2063	47
5	Queensland University of Technology	Australia	50	1566	41
6	Fudan University	Australia	50	717	39
7	Aston University	England	41	2181	32
8	Capital Medical University	China	41	752	24
9	Sun Yat-sen University	China	38	1988	26
10	University California, Berkeley	USA	36	1321	26

### Analysis of authors

**Fig. 5:**
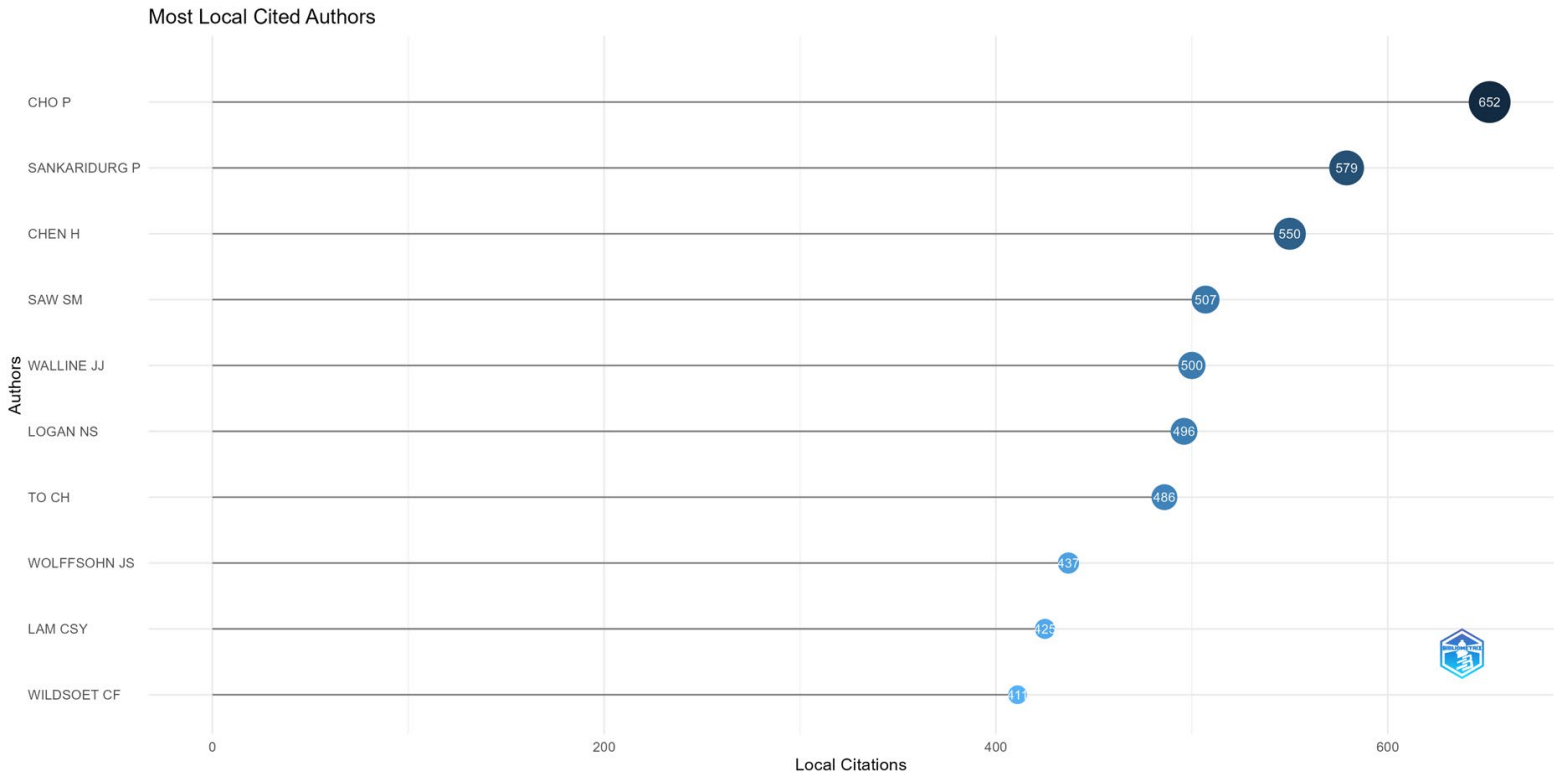
Top 10 most local cited authors

Author analysis data shows that a total of 3928 authors were involved, 69 fulfill more than 10 publications. 231 of them fulfill more than 5 publications. As shown in Fig. 5, the most local cited authors are Cho P, Sankaridurg P and Chen H. Local citations measure how many times an author included in this collection has been cited by the documents also included in the collection.

**Fig. 6:**
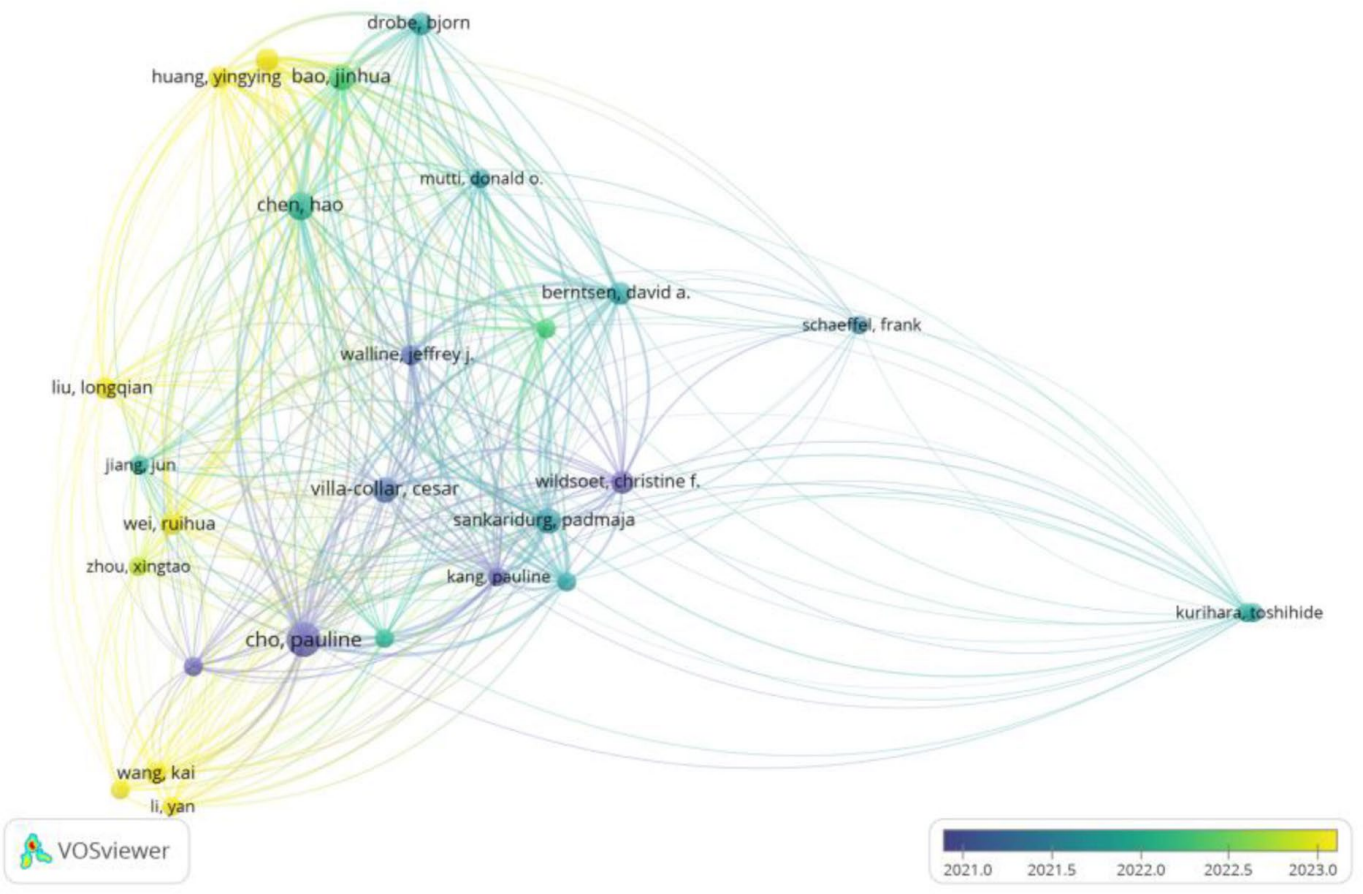
Time overlay of author collaborative networks and average publication of year with publication number greater than 15

Figure 6 illustrates the author collaboration network and average publication of year time overlay for authors of papers with no less than 15 publications in the field. Figures 5 and 6 showed that authors such as Cho P, Sankaridurg P and Chen H have likewise published relatively more articles and began publishing relatively early. The main areas of research are the two major segments of contact lenses and spectacles lenses. The highly cited core scholars all focus on the field of ortho-keratology [6, 7] and spectacle lenses [8–10]. Their papers clearly demonstrate the pivotal role of MMFL within the interdisciplinary field of clinical medicine, science and engineering based on co-word analysis.

### Analysis of publication source

**Fig. 7:**
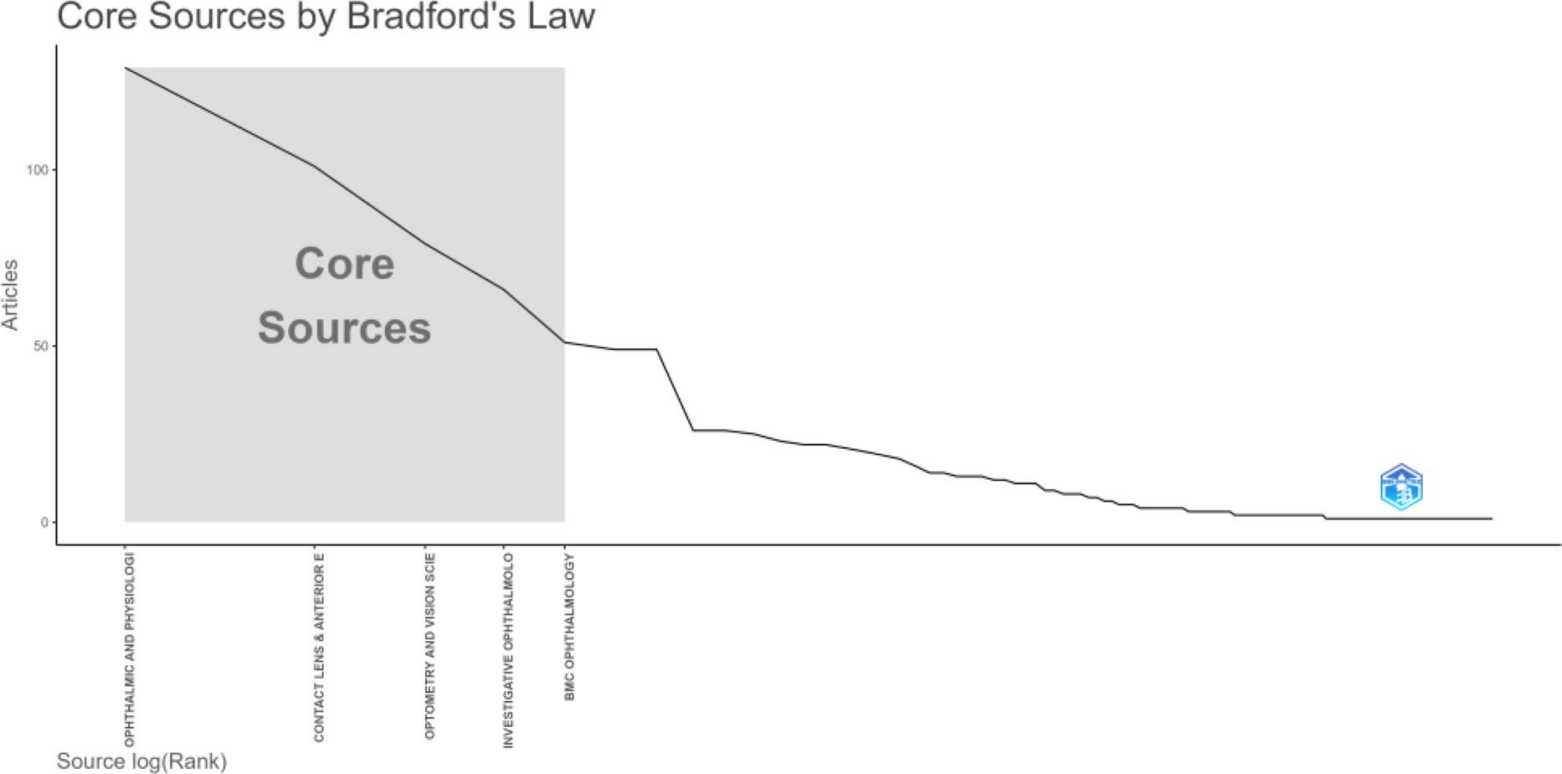
Core Sources by Bradford’s Law

The data shows that a total of 149 journals have published relevant literature. According to the core sources by Bradford's law (Fig. 7), the journals that published the most relevant articles are OPHTHALMIC AND PHYSIOLOGICAL OPTICS, CONTACT LENS & ANTERIOR EYE, OPTOMETRY AND VISION SCIENCE, all of which are the core authoritative journals in MMFL field, comprehensively elaborating MMFL from the mechanism of spectacle lens, contact lens-related physiological changes, changes in lenses fitting parameters, myopia prevention and control effects, and so on. The number of papers published in journals has increased over time.

**Fig. 8:**
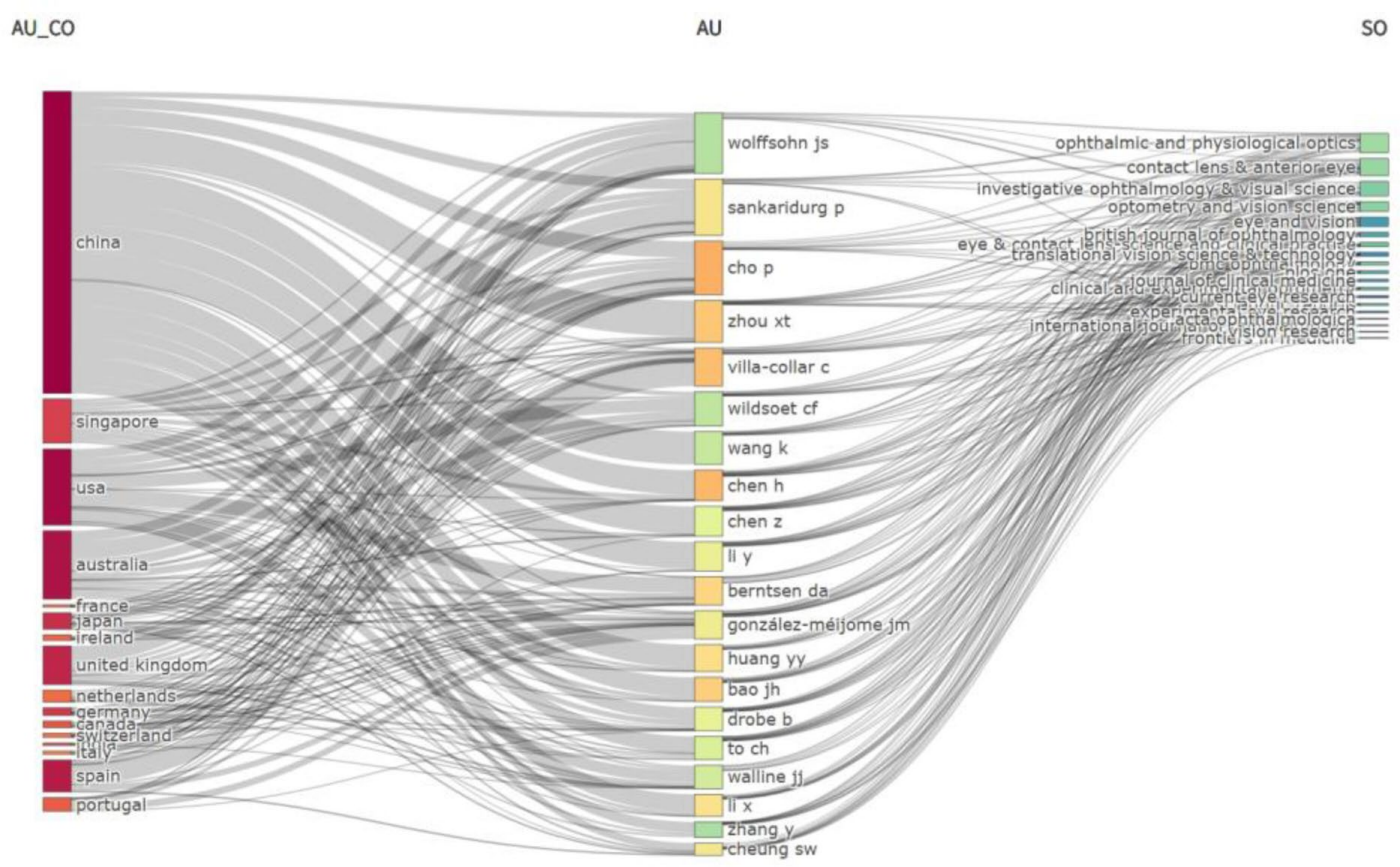
Country-Author-Journal three field plot

Country-Author-Journal three field plot visualizes the relationship between issuing countries, authors and journals (Fig. 8). The results show that countries with a high number of publications usually publish in journals with a high volume of literature and high impact, and that there is also a strong correlation between these journals and core authors.

### Analysis of citation

**Fig. 9:**
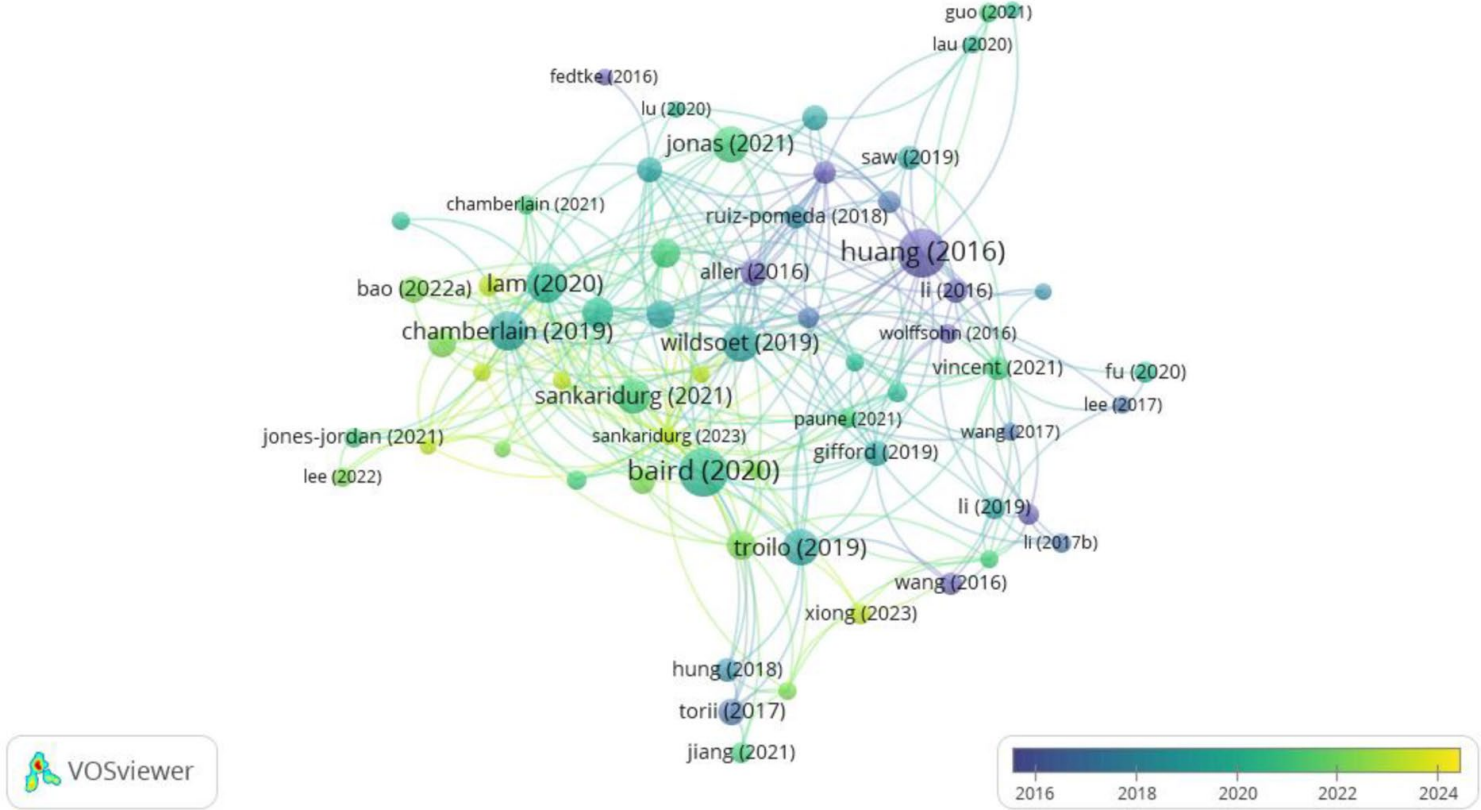
Time overlay of collaborative network of document citation. The warm color of the nodes indicating a later average publication time, with larger nodes meaning more frequently cited article

Figure 9 shows the 63 of 1143 articles with high citation frequency (publications are cited more than 60 times) were chosen for citation analysis, where the nodes of articles with high citation are relatively.

Table 2 shows the top 10 most cited paper, which were analyzed to reveal that the field of contact lenses and the field of spectacle lenses are the core areas of functional lenses research for myopia management. In particular, innovations in the optical field of functional lenses are the technological basis for supporting this field [11, 12], and the myopia management effects shown by the results of various clinical parameters are the key link in determining the utility of myopia management [13].

**Table 2:** Top 10 most cited paper

Rank	Paper	Total Citations	TC per Year	Normalized TC	Article’s name
1	HUANG JH, 2016, OPHTHALMOLOGY	550	55.00	11.65	Efficacy Comparison of 16 Interventions for Myopia Control in Children: A Network Meta-analysis
2	BAIRD PN, 2020, NAT REV DIS PRIMERS	542	90.33	14.81	Myopia
3	LAM CSY, 2020, BRIT J OPHTHALMOL	336	56.00	9.18	Defocus Incorporated Multiple Segments (DIMS) spectacle lenses slow myopia progression: a 2-year randomised clinical trial
4	CHAMBERLAIN P, 2019, OPTOMETRY VISION SCI	332	47.43	7.51	A 3-year Randomized Clinical Trial of MiSight Lenses for Myopia Control
5	TROILO D, 2019, INVEST OPHTH VIS SCI	306	43.71	6.93	IMI—Report on Experimental Models of Emmetropization and Myopia
6	WILDSOET CF, 2019, INVEST OPHTH VIS SCI	290	41.43	6.56	IMI—Interventions for Controlling Myopia Onset and Progression Report
7	JONAS JB, 2021, INVEST OPHTH VIS SCI	290	58.00	10.83	IMI Prevention of Myopia and Its Progression
8	SANKARIDURG P, 2021, INVEST OPHTH VIS SCI	262	52.40	9.79	IMI Impact of Myopia
9	WALLINE JJ, 2020, JAMA-J AM MED ASSOC	209	34.83	5.71	Effect of High Add Power, Medium Add Power, or Single-Vision Contact Lenses on Myopia Progression in Children: The BLINK Randomized Clinical Trial
10	JIANG Y, 2022, OPHTHALMOLOGY	195	48.75	10.19	Effect of Repeated Low-Level Red-Light Therapy for Myopia Control in Children: A Multicenter Randomized Controlled Trial

Among them, the paper titled "Efficacy Comparison of 16 Interventions for Myopia Control in Children: A Network Meta-analysis “ was the most cited paper with 550 citations [13], and defocus spectacle lenses was published by LAM CSY et al. in BRIT J OPHTHALMOL in 2020[12]. "Multiple Segments (DIMS) spectacle lenses slow myopia progression: a 2-year randomized clinical trial", was cited 336 citations. Literature with high citation frequency can effectively reveal the research hotspots and cutting-edge directions in this field.

### Analysis of keywords

**Fig. 10:**
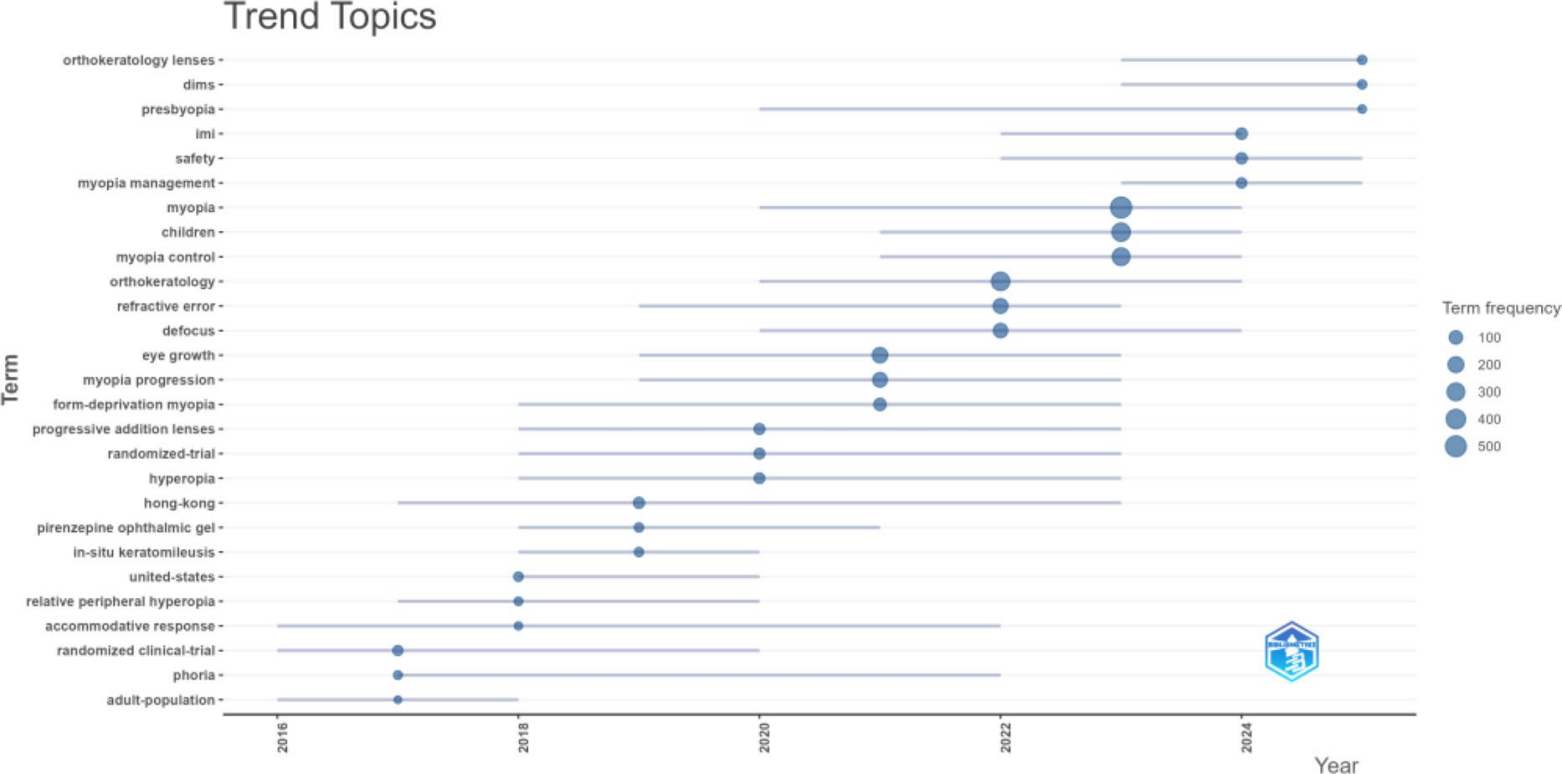
Trend topics (2016–2025)

Keywords provide an accurate summary of the core content of an article, reflecting the various topics covered by the literature and their interrelationships. This enables readers to quickly grasp the main research content and popular topics in a particular field of study, and gain a deeper understanding of its academic frontiers and research dynamics. As shown in Fig. 10, keywords show different thematic trends at different times. Ortho-keratology has a high term frequency. And “defocus” began to appear in 2020, mainly related to various types of defocused frame glasses and defocusing mechanism research.

#### Keyword co-occurrence analysis

**Fig. 11:**
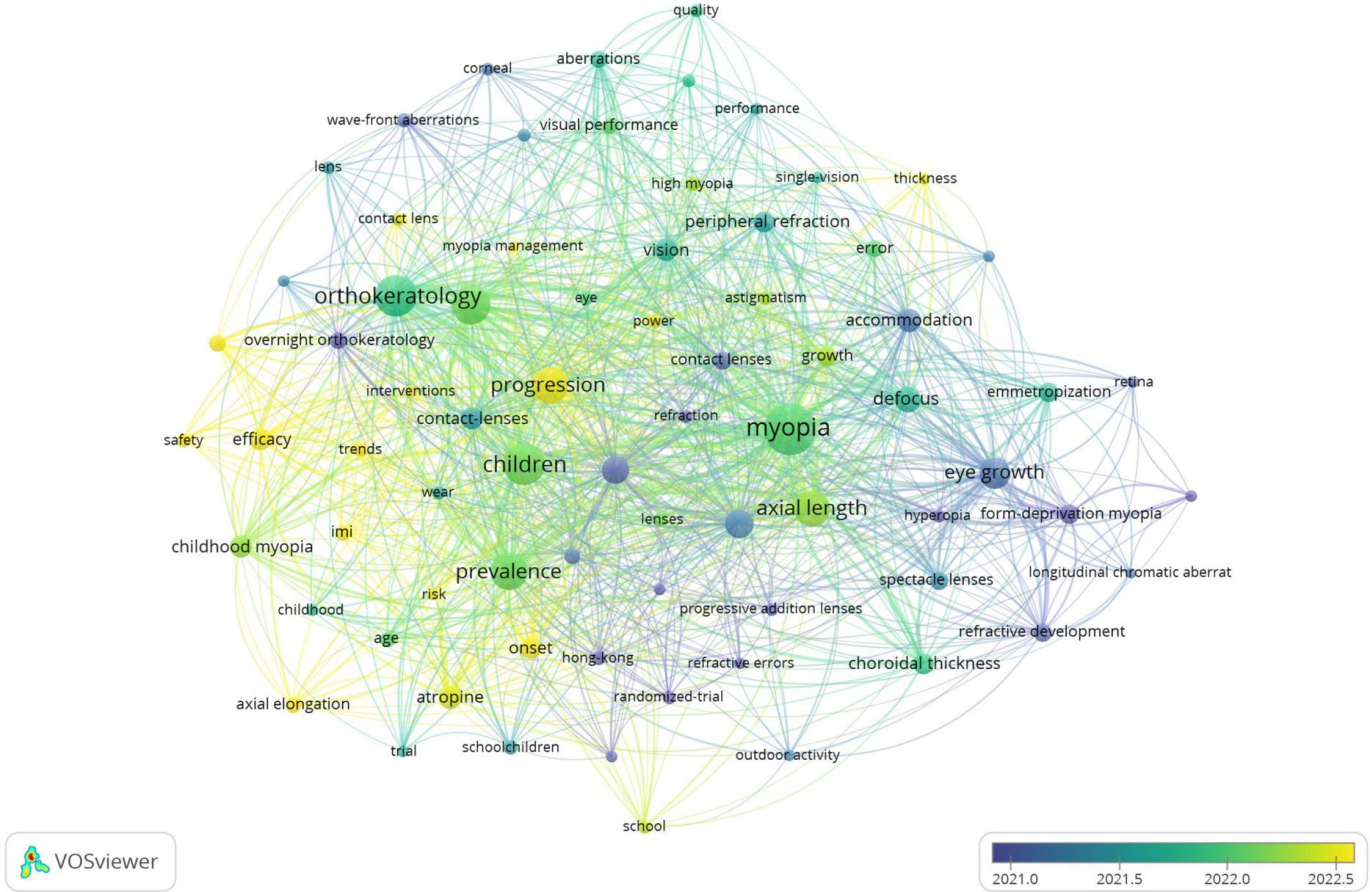
Time overlay visualization of Keyword co-occurrence

Figure 11 illustrates the co-occurrence (74 items) and the average time of occurrence for keywords with a frequency of not less than 25 occurrences. Unit of analysis mode is all keywords. It can be observed that due to different research focuses, keywords such as contact lenses, hydrogel and overnight orthkeratology, spectacle lenses appeared earlier, while axial length, onset and safety, interventions. Keywords such as myopia management, defocus have appeared frequently in recent years.

#### Keyword cluster analysis

Keyword clustering analysis can understand the knowledge structure and dynamic evolution of the field. Cluster number from large to small represents the number of nodes in the class from less to more. The more nodes, the more research hotspots.

**Fig. 12:**
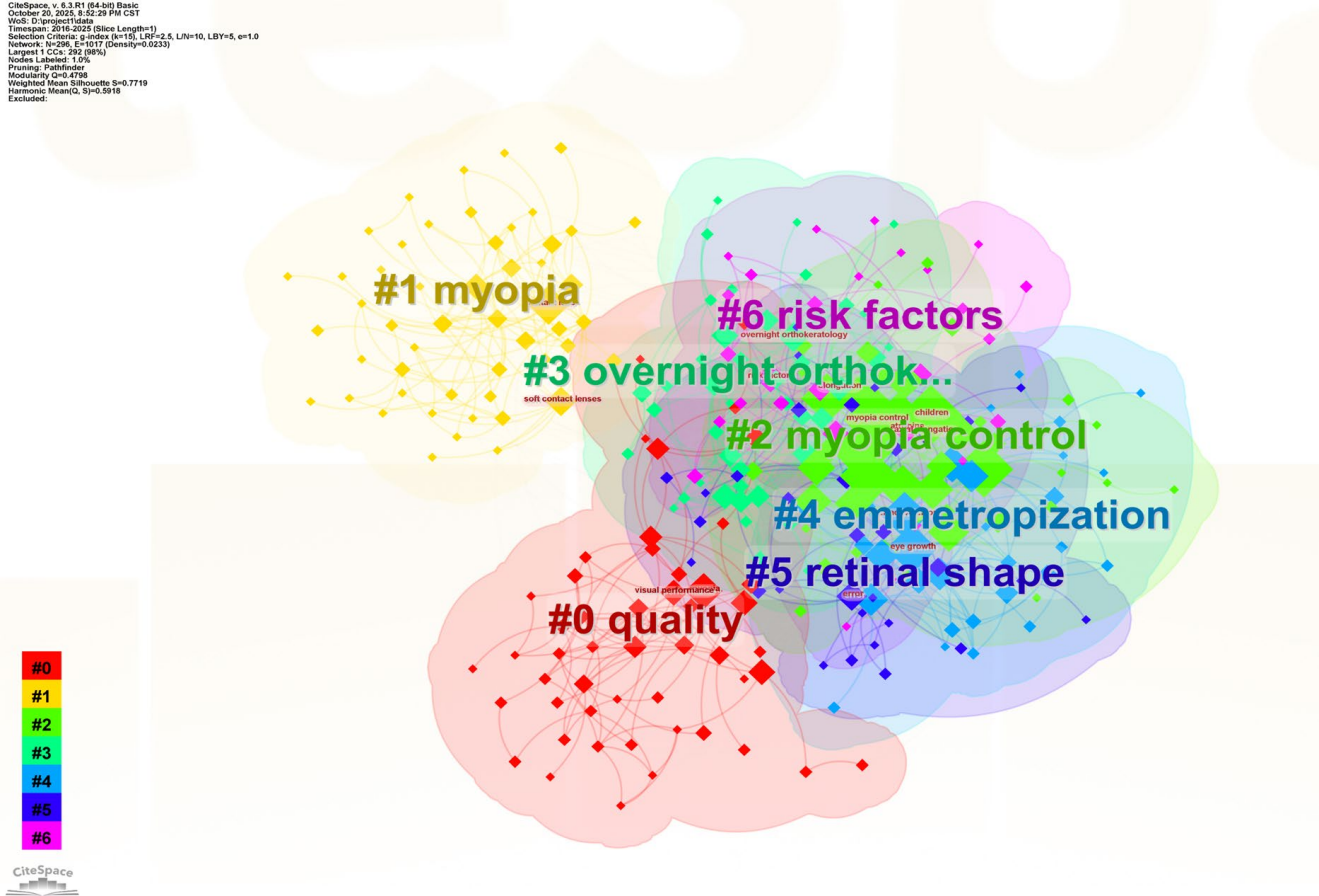
Visual mapping of keyword clustering of literature

The keywords are clustered and analyzed using CiteSpace software, and the keyword clustering map is generated, see Fig. 12. Q value = 0.4798 (> 0.3), which indicates that the clustering is effective, and S value = 0.7719 (> 0.7), which indicates that the clustering structure is significant and the clustering is reasonable. According to the potential semantic indexing algorithm, the keywords ranked by different clustering tags can be derived, see Table 3. The seven representative clustering tags were further summarized, analyzed, and concluded, which can be classified into two aspects: myopia management tools and myopia management utility observation. The tools aspect mainly focuses on the two categories of spectacle lenses and contact lenses. The myopia management utility mainly focuses on the axial length, diopter, visual acuity, and defocus parameters. These provide theoretical support and clinical practical effect tracking to determine the application evaluation and application improvement, thus promoting the innovation and development of the MMFL field.

**Table 3:** Topic keyword clustering of MMFL in the WOS core database

Cluster ID&NAME	Size	Silhouette	Mean (Year)	Label (LSI)
#0quality	51	0.74	2020	myopia control; multifocal soft contact lenses; high-order aberration; peripheral aberrations; resolution | myopia; vision; aberrations; lasik; visual function
#1myopia	51	0.863	2018	contact lens; water content; free water contact; h-1nmr methods; times relaxation | drug delivery; bacterial keratitis; silicone contact lenses; soaking method; misight contact lens
#2myopia control	47	0.855	2018	myopia control; axial length; myopia progression; long-term efficacy; choroidal thickness | contact lenses; myopia control spectacle lenses; harmon distance; accuracy; progressive addition
#3overnight ortho-keratology	45	0.736	2020	myopia control; order aberrations; pupil diameter; contrast sensitivity; motion perception | axial length; contact lens; visual acuity; computer-aided fitting; artificial intelligence
#4emmetropization	34	0.777	2019	axial length; form deprivation; choroidal thickness; spectacle lenses; experimental myopia | refractive error; eye growth; accommodation; prevalence; spherical aberration
#5retinal shape	33	0.669	2020	myopia control; contrast sensitivity; motion perception; peripheral vision; visual performance | axial length; retinal shape; single vision; error; emmetropia
#6 risk factors	31	0.704	2019	prevalence; children; progression; visual impairment; accommodative lag | myopia progression; multifocal contact lens; discontinuation o; misight contact lens;

In Table 3, based on (Latent Semantic Indexing, LSI) technology [14], Cluster ID#0 Quality reflects the importance of lens utility is myopia management, including lens control effects. Cluster ID#1myopia covers a wide range of methods involved in myopia management, including drug delivery. Cluster #3 overnight ortho-keratology is an important branch of functional lenses for myopia management, lens material innovation is an important concern. Cluster #4 emmetropization. Axial length is an important effect performance parameter in myopia management. Embodying Clinical Medicine and Biology. Cluster#5 retinal shape. Scleral Parameters and scleral remodeling have been the focus of research in recent years, targeting the sclera to study peripheral refraction and exploring the biological mechanisms of myopia progression. Cluster#6 risk factors. Various types of ocular bio-parameters of ocular growth progression, including the causes and dangers of various alterations in the occurrence of judgment and prognosis.

#### Keyword bursts analysis

**Fig 13:**
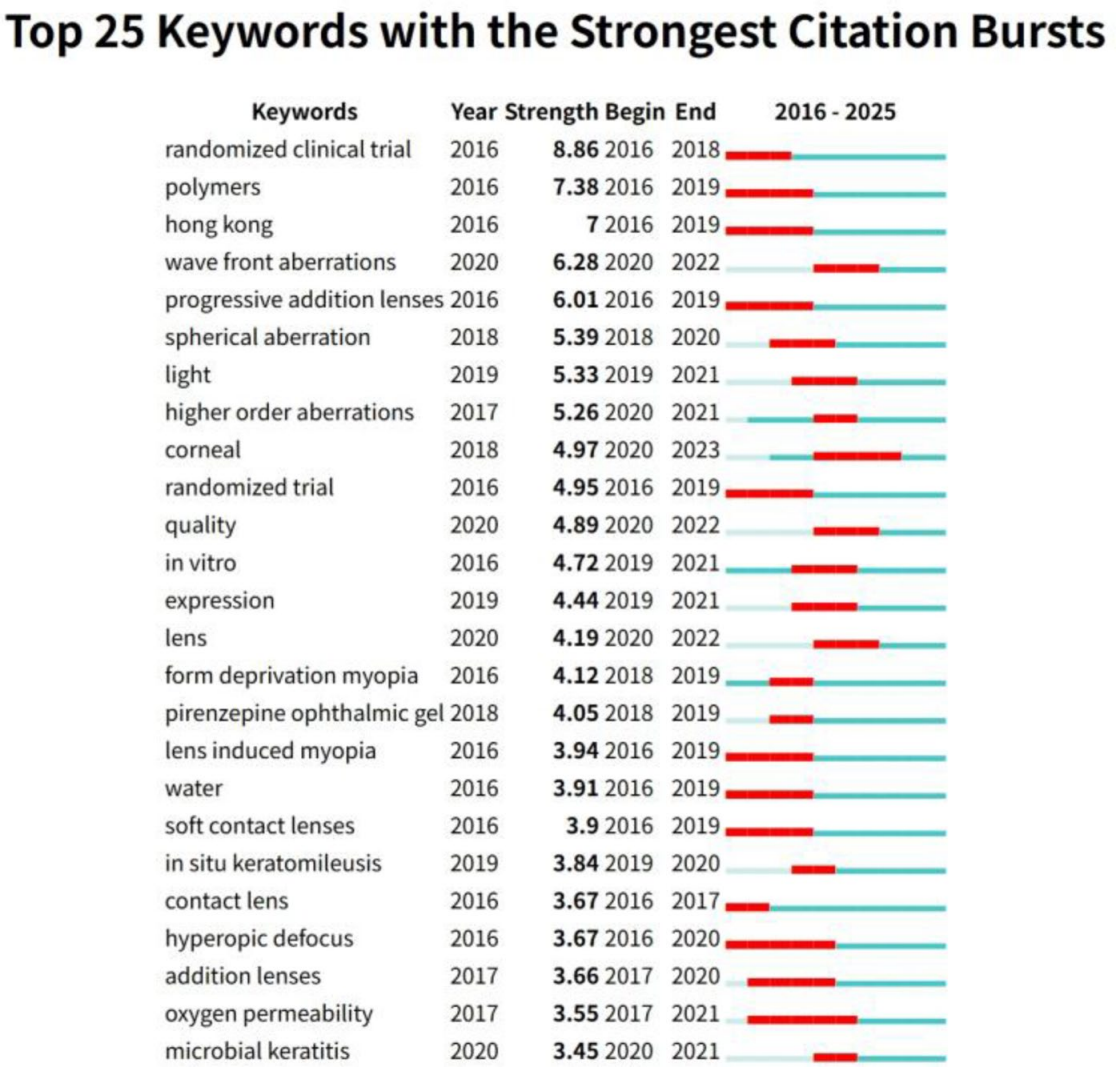
Top 25 with the strongest citation bursts keywords. The time period in which the node has not appeared will be marked with a transparent blue color, the time period in which it has appeared will be marked with an opaque blue color, and the bursts time period in which it has appeared will be marked with a bolded red segment markers

Through keyword bursts analysis, keywords with high frequency change rate and fast growth rate in a certain period of time are calculated to reveal the research hotspots and development trends in the field, and 25 keywords with the highest burst intensity are detected, see Fig. 13.

In 2016–2018, randomized clinical trial (efficacy validation of myopia management), polymers (material aspects), and Hong Kong (actually the place where DIMS lenses was invented) were the research themes that emerged one after the other. The emergent words for technical aspects include wave front aberrations, progressive addition lenses, spherical. Indicating that the focus of research at this stage is on the optical aspects of the lenses. 2019 saw the addition of new research themes such as light, quality, and pirenzepine ophthalmic gel and other research topics. This phase of research focuses on optical and pharmacological means of combined myopia management, and explores the effects of multiple myopia prevention and control means, indicating that researchers are beginning to combine the use of medication and functional glasses from clinical and management perspective. Hyperopic defocus, addition lenses, and oxygen permeability, which became a new research hotspot in 2016, include randomization, randomization, and randomization of lenses. Research hotspot, of which, the randomized clinical trial has a high emergent strength of 8.86, which is the core of the MMFL research in this decade. At this stage, the research focuses on the clinical application effect of functional lenses and explores the principle of defocus. In particular, the clinical effect validation studies of multiple defocus frame spectacles and defocus soft corneal contact lenses. In addition, emerging technologies such as defocus spectacles [8, 12] and soft contact lenses [11] also play an important role in the development of MMFL. The prominence of the above keywords reflects the wide application and rapid progress of multidisciplinary technologies.

## Discussion

### Current situation discussion

The prevalence of myopia among children worldwide is on the rise, and high myopia is associated with complications that can lead to vision loss in adulthood. During adolescence, assisting parents in managing myopia is a key responsibility of optometrists and other professionals in the prevention and control of myopia. The “Healthy China 2030” Outline proposes “strengthening early diagnosis, early treatment, and early rehabilitation across all populations and throughout the entire life cycle,” with its core objective being to advance national health through the integrated prevention-treatment-rehabilitation continuum. In the field of visual health, it is essential to popularize visual health services and maintain and safeguard visual health comprehensively and throughout the entire life cycle. At the policy level, China has clarified the direction of technological research through documents such as the “Brightness Action Plan for Myopia Prevention and Control in Children and Adolescents (2021–2025).” In 2025, the Ministry of Education in china further proposed “guidelines for myopia prevention and control at different educational stages, including preschool, primary school, and secondary school,” focusing on promoting the standardization of emerging fields such as functional lenses for full-cycle myopia prevention and control. In rapidly advancing technological domains: Teams led by Bao Jinhua [15]and Jiang Jun [16]at Wenzhou Medical University innovate functional lenses; Chen Zhi's team in Shanghai explores pre-myopic functional lenses [17], optical films on single-vision spectacles [18] and Artificial intelligence-assisted fitting method in orthokeratology lens fitting [19]; The Beijing-based team led by Wang Kai is researching intelligent orthokeratology lenses [20]. Various myopia management policies have spurred the development of research on functional eyewear for myopia prevention and control.

Keyword analysis indicates that the design and application of myopia management lenses currently represent a highly interdisciplinary field, encompassing medicine, biology [21], management science [22], engineering [23], and other disciplines. Research in the field of visual health concerning myopia management lenses primarily covers their impact on ocular development and growth, lens materials [24], innovative lens designs, and their outcomes [15, 25] and mechanisms in controlling myopia progression. Overall, myopia management lenses serve multiple purposes, from influencing ocular development and refractive status to functioning as tools for myopia control and visual health.

In keyword bursts analysis, drug delivery; bacterial keratitis; myopic contact lens; computer-aided fitting; artificial intelligence; emmetropia; signaled that the field of myopia management is moving toward disciplinary cross-fertilization proceeding through materials, smart technologies and medical applications. The emergence of high-frequency keywords and clustering characteristics reveal the stage-by-stage evolution of technology iterations not only demonstrates the rapid progress in the field of functional lenses for myopia management, but also the intertwining and innovation of multidisciplinary technologies.

Today, progress in MMFL is drawing attention from a wide range of people. On the one hand, the results of bibliometric analysis will play a significant role in the public health management of myopia prevention and control. Optometrists and various types of managers can use literature searches to understand the statistical characteristics of communities and patient populations, the social determinants of health, and epidemiology, thereby gaining insight into current trends regarding visual health, functional lenses, and, in particular, functional lenses related to myopia prevention and control. Additionally, they can gain an understanding of hot topics closely related to functional lenses for myopia management, such as the eye, vision, healthcare, and health literacy. On the other hand, this has significant implications for various non-medical professionals involved in myopia management, including parents, teachers, government officials, and practitioners in the functional lenses industry. This article will help them understand the overall landscape of myopia management lenses, grasp the current state of the industry, and plan for future development in alignment with relevant policies.

### Exploration of multidisciplinary development trends

#### Optical materials

In terms of material research and development, various new optical materials have been developed, such as innovative changes in materials for contact lenses for myopia management, improvement in oxygen permeability of hydrogel [24], low concentration atropine drug sustained-release lenses [26], modification of the surface coating of contact lenses, and improvement of materials for contact lens and spectacle lenses, and other innovative optical designs. Advancements in nanotechnology have enabled contact lenses [27] to be used as drug delivery devices, opening up further opportunities.

Key advances in optical material science will be the core driver for the development of functional eyewear for myopia management. Natural biomaterials, derived from renewable biological sources, offer significant advantages including biocompatibility, biodegradability, minimal immunogenicity, wettability, and favorable patient comfort [28].

For contact lenses, high oxygen permeable polymers, novel hydrogels, liquid crystal materials, and optical nanomaterials offer future breakthroughs in improving corneal health, optical performance, and wearing experience. Changes in material oxygen permeability reduce usage complications, support the design of thicker and more optically complex lenses through material evolution without sacrificing ocular health. Material innovations and surface treatment technologies have improved comfort and stability. The impact enhance lenses comfort and wearer compliance, reduce contact lens protein and lipid deposits, and keep the lens surface moist. In the spectacle lens dimension, the lenses itself maintains the stability of the optical properties, various functional coatings keep the optical interface of the lens clean over time, ensuring continuity and precision of optical effect and fewer deposits of surface.

In the field of future myopia management and optical materials research, current findings may affect the design of future clinical trials, sofurther studies are needed to determine the optimal duration and concentration of atropine for controlling childhood myopia [29, 30]. On smart invisible shield aspect, colorless, durable and self-defending contact lens nanocoating maybe used in myopia management functional lens [31]. Future research should prioritize the development of robust biometric chemical technologies, wireless optical reading technologies, and scalable manufacturing strategies to support clinical translation. Contact lens biosensors are set to play a pivotal role in the next generation of personalised healthcare by enabling non-invasive analysis of tear fluid [32]. The innovative prototype of the eye-machine interaction contact lens goes beyond the capabilities of traditional brain-computer interfaces [33].

Future research should also focus on biomimetic interface engineering. Examples include cornea-mimicking nanostructures and AI-driven dynamic optimization, such as causal network-regulated drug release. Multidisciplinary approaches combining gene editing with smart materials should also be explored [34]. In functional lenses, Particular focus is placed on smart coatings that respond to stimuli such as pH, enzymes and radiation, and on nanocellulose-based systems as sustainable, tunable platforms [35].

#### Optical engineering

Research on improving lenses in various optical designs is currently underway [36, 37]. In the field of optical engineering, Exploring the optimization of optical design and its effects on myopia management outcomes, including the efficacy of single-design approaches versus combined-design approaches, and the integrated application of medical optometry technologies such as optical management. Moreover, the selection of light sources for myopia management is also an important area of research. Latest research indicate repeated low intensity red light is a safe, non-invasive option for managing extreme myopia in children, with meaningful clinical value [38].

As we all know, contact lenses that slow the progression of myopia incorporate concentric rings of plus power, a peripheral optical zone with add power, or non-monotonic variations in power [39]. In the future, various optical design spectacle lens will also be invented. Optical engineering aspects of MMFL will be widely used. Further exploration of optical design optimization and optical management effects will be conducted in the future.

#### Artificial intelligence (AI)

Artificial intelligence will be extensively integrated with myopia management lenses and techniques [40]. For instance, it identifies myopia risk parameters by analyzing pre- and post-use data from functional lenses to assess progression risks, screen potential influencing factors, and develop personalized management plans. Children and adolescents identified as high-risk should promptly receive tailored myopia control solutions. Currently, the role of computer artificial intelligence in determining parameters for myopia management lenses primarily includes: Determining relevant parameters for orthokeratology lenses; Combining defocus parameter measurements to determine parameters for spectacle lenses or contact lenses; AI also plays a role in predicting the therapeutic efficacy of myopia control lenses and identifying influencing factors. East Asia is well-positioned to pioneer a comprehensive, equitable digital model for myopia control in the future [41], Integrating biometric and multimodal imaging data for early prediction of myopia onset.

#### Medicines

Low-concentration atropine, various designs of peripheral defocus spectacles, contact lenses, and RLRL effectively slow myopia progression [42, 43]. On one hand is the efficacy of monotherapy itself, and on the other is the efficacy of combination therapy. Whether these modalities used together have an additive, antagonistic, or multiplicative effect awaits further exploration.

#### Management

Full Stage use of MMFL is an important issue in the future [1]. The optometric affiliations has established a closed-loop health management system of “prevention-diagnosis-treatment-rehabilitation”, which realizes comprehensive monitoring and full intervention of the visual health with functional lens fitting, and carries out refined visual health management. Research on multifunctional eyewear industry management will become a vital aid in visual health management in the future. The cost-effectiveness of myopia-control functional lenses for the reduction of myopia progression among children and adolescents will be assessed thoroughly [30].

#### Discussion on future trends

The development path of functional lenses for myopia management ranges from key technology breakthrough to wide application, and the technological development shows the stage characteristics of “basic breakthrough—performance optimization—scene landing”. The basic breakthrough and performance optimization are mainly the key technology research, including the design of spectacle lenses and contact lenses, the modification of the material membrane layer, and the development and enhancement of drug release technology; and the scenarios on the ground are mainly reflected in the medical scenarios such as light therapy, atropine therapy, and visual rehabilitation training, and the joint application. Based on the keyword clustering and emergent strength analysis, the application of functional lenses in visual health presents two major characteristics of “precision and personalization”, covering multiple scenarios pre, current and post- myopia management.

Nowadays, optical correction method, pharmacological, light therapy and behavioral interventions for slowing myopia progression in children. MMFL is an important optical measure, and also is one of the preferred myopia management tools for optometrists. In the future, studies are also crucial for gaining a more comprehensive understanding of how various environmental and lifestyle factors can be modified to prevent or slow the progression of myopia [44]. Evidence suggests that higher levels of screen time are associated with an increased risk of various health issues. Future studies also should investigate the specific effects and mechanisms of screen time on health [45].

The future development of functional lenses for myopia management presents three major features: multidisciplinary intersection, material innovation and optical innovation with intelligent application. Functional lenses will be extended from single device innovation to “hospital-community-family” three-level service system, and multidisciplinary crossover and integration will provide systematic solutions for myopia management. Under the background of visual health, the integrated development trend of “material-optical-intelligent” will lead the research of functional lenses for myopia management. Moreover, Research into the development of MMFL must also consider various social and cultural factors in the public health sector [46–49].

## Challenge

At present, although MMFL show great potential, they still face many challenges. safety and stability of optical materials are insufficient, there is an urgent need to develop lens materials, such as myopia management contact lenses related to hydrogel, oxygen permeability, antibacterial and drug release function; Although the technology of defocusing technology is becoming more and more mature, but how to enhance the effect of defocusing without affecting the performance of the daily correction system is still a difficult problem to be solved; How to scientifically and objectively evaluate the association between the defocus and myopia prevention and control effects; How to improve the correction accuracy and the speed of correction response effect. In the end, the lack of unified evaluation standard in the field of functional lenses, it is urgent to formulate related standards and expert consensus to ensure its reliability, safety and clinical value.

## Limitations

The literature cited in the discussion section of this paper does not derive entirely from the results of this bibliometric search. On the one hand, the initial keywords may have led to certain omissions during the screening process; on the other hand, these discussions were written by our team based on the results of this bibliometric search, combined with our assessment of future professional trends. Furthermore, our assessment and judgment of future trends in MMFL may be influenced by the cognitive limitations of our team.

## Conclusion

This study is the first to utilize bibliometric analysis to construct a knowledge map on MMFL in the field of visual health, revealing the current state of research trend. Future research will span multiple disciplines including optical materials, optical engineering, AI, medicines, and management, exhibiting three key characteristics: multidisciplinary integration, precision, and personalization. The integrated development trend of “materials-optics-intelligence” will guide MMFL research, propelling these products toward intelligent, multifunctional designs and broad clinical applications.

## Data Availability

Data are available on request.
